# Regulation of SIRT1 and Its Roles in Inflammation

**DOI:** 10.3389/fimmu.2022.831168

**Published:** 2022-03-11

**Authors:** Yunshu Yang, Yang Liu, Yunwei Wang, Yongyi Chao, Jinxin Zhang, Yanhui Jia, Jun Tie, Dahai Hu

**Affiliations:** ^1^ Department of Burns and Cutaneous Surgery, Xijing Hospital, Fourth Military Medical University, Xi’an, China; ^2^ Department of Emergency, Xijing Hospital, Fourth Military Medical University, Xi’an, China; ^3^ State Key Laboratory of Cancer Biology and Xijing Hospital of Digestive Diseases, Xijing Hospital, Fourth Military Medical University, Xi’an, China

**Keywords:** SIRT1, gene regulation, enzyme activity, post-translational modification, inflammation

## Abstract

The silent information regulator sirtuin 1 (SIRT1) protein, a highly conserved NAD^+^-dependent deacetylase belonging to the sirtuin family, is a post-translational regulator that plays a role in modulating inflammation. SIRT1 affects multiple biological processes by deacetylating a variety of proteins including histones and non-histone proteins. Recent studies have revealed intimate links between SIRT1 and inflammation, while alterations to SIRT1 expression and activity have been linked to inflammatory diseases. In this review, we summarize the mechanisms that regulate SIRT1 expression, including upstream activators and suppressors that operate on the transcriptional and post-transcriptional levels. We also summarize factors that influence SIRT1 activity including the NAD^+^/NADH ratio, SIRT1 binding partners, and post-translational modifications. Furthermore, we underscore the role of SIRT1 in the development of inflammation by commenting on the proteins that are targeted for deacetylation by SIRT1. Finally, we highlight the potential for SIRT1-based therapeutics for inflammatory diseases.

## Introduction

Members of the sirtuin (SIRT) family of proteins are class III histone deacetylases (HDAC III) that are homologous to yeast silent information regulator 2 (Sir2). Sirtuins mediate the deacetylation of histones and non-histone proteins in an NAD^+^-dependent manner (1). SIRT1 was the first SIRT to be discovered in mammals; it shares the highest homology with Sir2, and is the most extensively-studied SIRT protein that plays a role in promoting longevity (2). SIRT1-mediated deacetylation profoundly impacts multiple biological processes, including cellular senescence (3), apoptosis (4), sugar (5) and lipid (6) metabolism, oxidative stress (7, 8), and inflammation (7). Thus, even minor changes in SIRT1 expression and function can significantly impact cellular responses.

SIRT1 is well known for its antioxidant and anti-inflammatory properties (7–9). As such, SIRT1-targeted anti-inflammatory therapies are attracting increasing attention for their clinical applications in treating inflammatory diseases (10). In addition, several signaling pathways provoked by immune cell activation are tightly associated with SIRT1 function. This review focuses on the upstream and downstream regulators of SIRT1 expression and function and the roles of SIRT1 in inflammation.

## SIRT1 Protein Structure

The human *SIRT1* gene is located on chromosome 10q22.1, contains 9 exons and 8 introns, and encodes a protein composed of 747 amino acid (aa) residues, whereas murine *SIRT1* encodes 737 aa residues. SIRT1 is ubiquitously expressed in multiple human tissues and cells, and its subcellular localization varies depending on the tissue or cell type, stress level, and interaction with other molecules. The SIRT1 protein contains N-terminal, catalytic, and C-terminal domains. In terms of its three-dimensional structure, SIRT1 is composed of a major Rossmann-fold domain that is highly conserved, and a minor domain containing a zinc-binding module and a helical module. Catalytic reactions are initiated by the binding of the acetylated residue of the target molecule and NAD^+^ *via* the cleft between these two domains (1).

## Chemical Process of Protein Deacetylation by SIRT1

**Figure 1:**
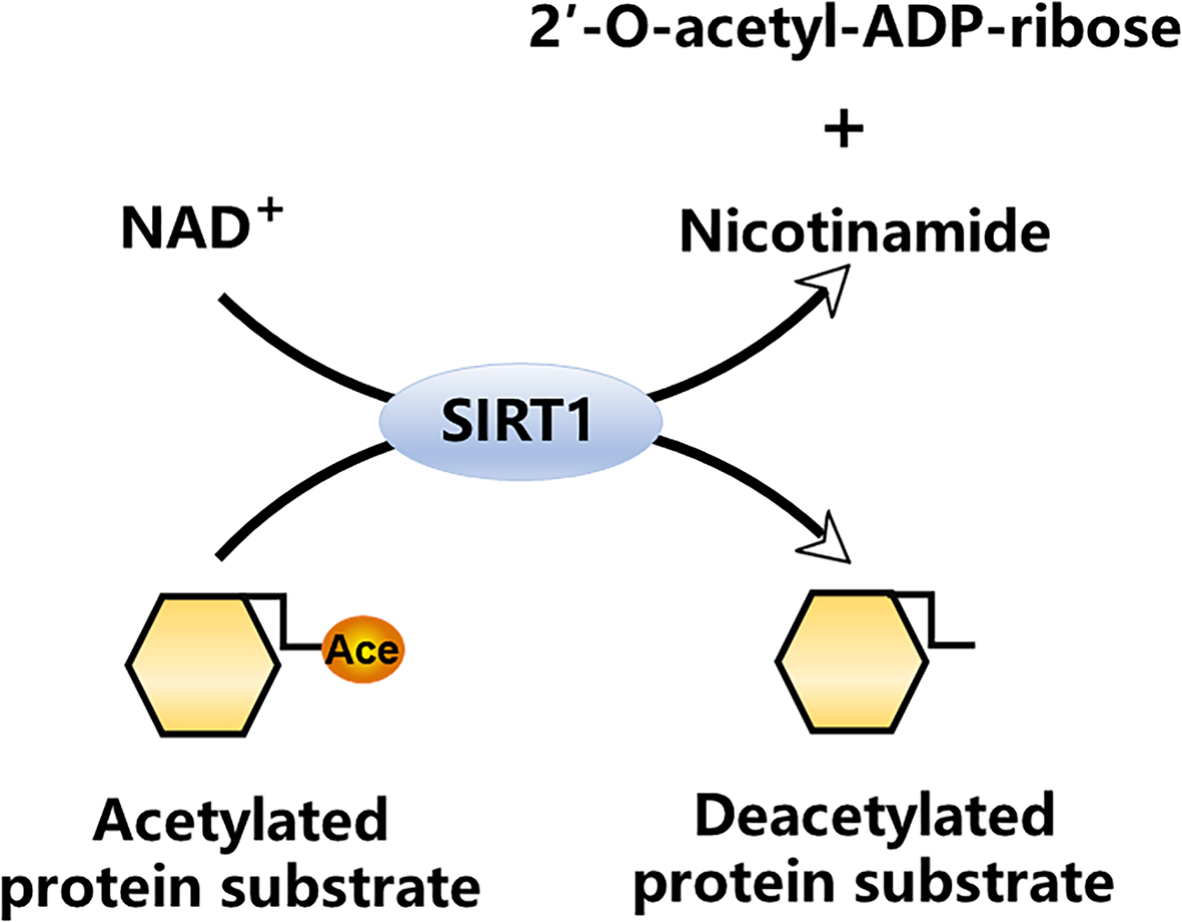
NAD^+^-dependent SIRT1 deacetylase reaction.

SIRT1 is dependent on NAD^+^ for catalysis. SIRT1 deacetylates target proteins by hydrolyzing NAD^+^ and simultaneously transferring the lysine-bound acetyl group from acetylated proteins to the 2´-OH position of ADP-ribose, ultimately yielding nicotinamide and 2´-O-acetyl-ADP-ribose (**Figure 1**) (11, 12). The NAD^+^ dependence determines that the levels of NAD^+^ and SIRT1 activity are tightly coupled.

## SIRT1-Mediated Deacetylation of Target Proteins

**Figure 2:**
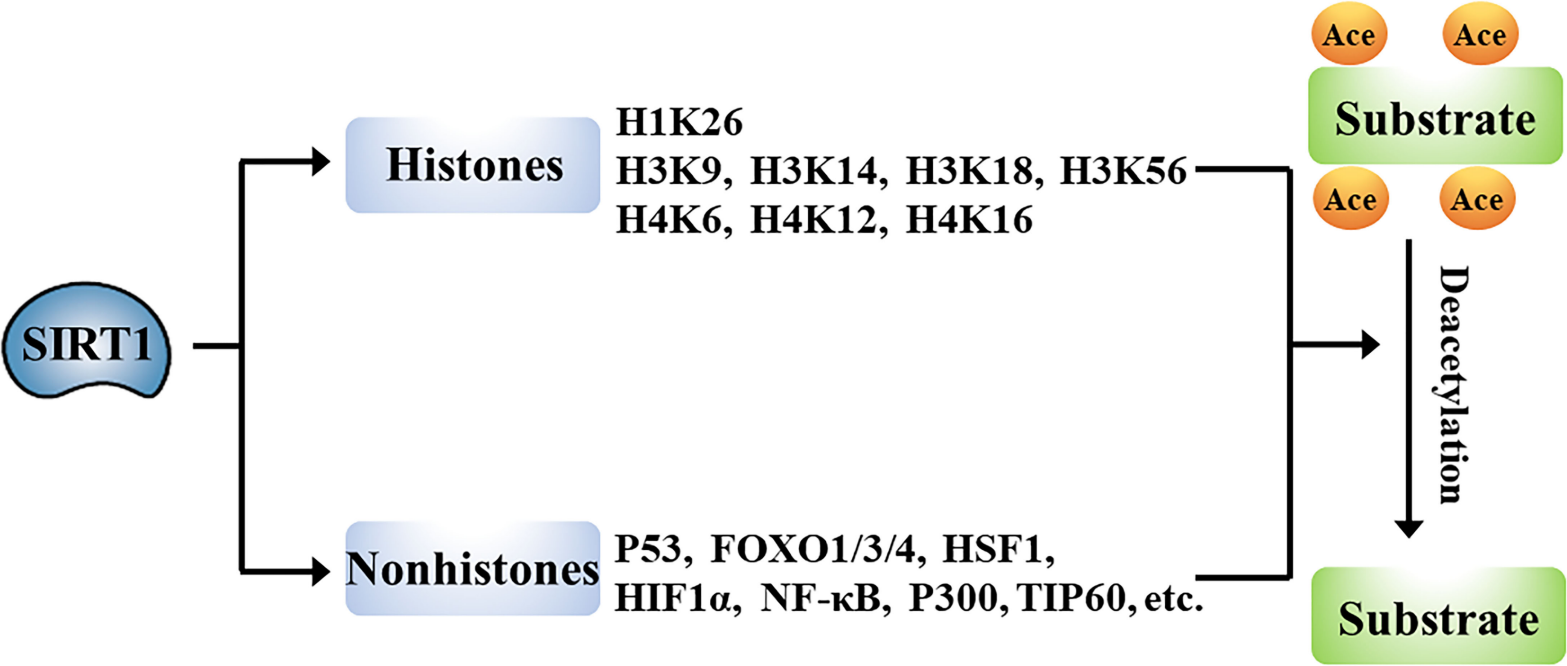
Schematic overview of the genes targeted for deacetylation by SIRT1.

The wide-ranging biological roles of SIRT1 are largely mediated by its functions in deacetylation of target proteins that can include histones or non-histone proteins (**Figure 2**).

### Histones

Histones are chromatin-associated proteins in eukaryotic cells that function to condense DNA into compact shapes known as nucleosomes, which play essential roles in the maintenance of chromosome conformation and regulation of gene transcription. Acetylation of N-terminal lysine residues of histone proteins can directly influence gene transcription. Numerous studies have shown that SIRT1 deacetylates histones. For example, SIRT1 deacetylates lysine 26 of histone H1 (H1K26); lysine 9, lysine 14, lysine 18, and lysine 56 of histone H3 (H3K9, H3K14, H3K18, and H3K56); and lysine 6, lysine 12, and lysine 16 of histone H4 (H4K6, H4K12, and H4K16) (13, 14). SIRT1-mediated deacetylation of promoter-associated H3K9 and H4K16, and subsequent suppression of transcription have been particularly well characterized (15–17).

### Non-Histones

SIRT1-driven deacetylation of multiple non-histone proteins has been shown to impact pathophysiological processes such as cell differentiation (18), apoptosis (4), autophagy (3), metabolism (6), and inflammation (7). SIRT1 can directly deacetylate multiple transcription factors or co-factors including P53, forkhead-box transcription factor 1/3/4 (FOXO1/3/4), heat shock factor 1 (HSF1), hypoxia-inducible factor 1 alpha (HIF-1α), nuclear factor kappa B (NF-κB), P300, and TIP60, to regulate the transcription of their target genes (19–23). Alternatively, SIRT1 has been shown to indirectly promote the function of transcription factors, including peroxisome proliferator-activated receptor α/γ (PPARα/γ), myoblast determination protein (MyoD), and others. Additionally, SIRT1 directly deacetylates many non-transcriptional regulatory proteins, including autophagy-related protein (ATG) and liver kinase B1 (LKB1) (24, 25). The deacetylase activity of SIRT1 thus impacts multiple biological processes.

## Regulation of SIRT1 Expression

**Figure 3:**
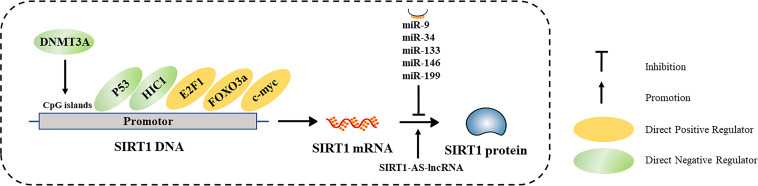
Characterized mechanisms that regulate SIRT1 expression, including transcriptional regulation and post-transcriptional regulation.

SIRT1 expression is subject to many layers of regulation, including at the transcriptional and post-transcriptional (**Table 1** and **Figure 3**). In this section, we discuss the diverse cellular mechanisms that contribute to the regulation of SIRT1 expression at each of these levels.

**Table 1:** Regulation of SIRT1 expression.

Level of Regulation	Specific Mode	Molecules	Effect
Transcriptional regulation of SIRT1	DNA methylation	DNMTs	Downregulation
	Transcription factors	P53, HIC1	Downregulation
		E2F1, FOXO3a, c-myc	Upregulation
Post-transcriptional regulation of SIRT1	ncRNAs	miR-9, miR-34, miR-133, miR-146, miR-199	Downregulation
		SIRT1-AS-lncRNA	Upregulation
	HuR RNA-binding protein	HuR	Upregulation

### Transcriptional Regulation of *SIRT1*

#### DNA Methylation

DNA methylation is an important epigenetic modification that regulates gene transcription. DNA methyltransferases (DNMTs) methylate specific nucleotide sequences, especially CpG islands in gene promoters (26). In general, hypermethylation of CpG islands in promotor regions is negatively correlated with gene transcription. DNA hypermethylation may be associated with reduced SIRT1 expression in some diseased tissues. For example, Islam et al. found that SIRT1 was hypermethylated in oral epithelial cells of patients with oral cancer who chew areca, and confirmed that arecoline induces hypermethylation and subsequent downregulation of SIRT1 *in vitro* (27). Chen et al. discovered that SIRT1 expression in the heart was significantly decreased in a fetal rat model of gestational diabetes compared to that of hearts in control rats (28). *In vitro* treatment of rat hearts with the DNA methylation inhibitor 5-azacytidine (5-AZA) inhibited DNMT3A, which consequently increased SIRT1 protein levels. Thus, *SIRT1* transcription is impacted by DNA methylation.

#### Regulation of SIRT1 by Transcription Factors

*SIRT1* transcription is regulated by a variety of transcription factors and co-factors including P53 and hypermethylin cancer 1 (HIC1), which repress *SIRT1* transcription, and E2F1, FOXO3a, and C-MYC which promote *SIRT1* transcription. *SIRT1* transcription is also regulated by negative feedback loops, which results in steady state *SIRT1* transcript levels varying dynamically across different cellular states.

##### P53

The acetylation of P53 is known to affect its transcriptional activity; SIRT1 directly deacetylates lysine 382 of P53. In addition, P53 can form a negative feedback loop that inhibits *SIRT1* transcription by binding to the P53 response element in the *SIRT1* promoter (29, 30).

##### HIC1

HIC1 participates in the negative regulation of *SIRT1* transcription. Chen et al. found that hypermethylation of HIC1 reduced its expression in tumor cells, leading to dramatic upregulation of *SIRT1* expression (31). HIC1 binds to SIRT1 and C-terminal binding protein 1 (CTBP1), forming an inhibition complex that represses SIRT1 expression by interacting with enhancer elements upstream of the *SIRT1* promoter (32). Additionally, SIRT1 and HIC1 can participate in negative feedback loops. Stankovic-Valentin et al. showed that both SIRT1 and HDAC4 could interact with HIC1, mediating the deacetylation of HIC1 at lysine 314 and concurrently promote SUMOylation, inhibiting the transcriptional activity of the HIC1 complex (33).

##### E2F1

The E2F1 transcription factor interacts with two binding sites in the *SIRT1* promoter to induce *SIRT1* transcription, and SIRT1 can inhibit its own transcription by deacetylating E2F1 (34). Wang et al. identified that this negative feedback loop is important to the regulation of apoptosis in response to DNA damage (35). The E2F1/P73 pathway is thought to play an essential role in DNA-damaging drug-induced apoptosis (36). Pediconi et al. demonstrated that SIRT1, PCAF, and E2F1 are co-recruited on the P73 promoter, and SIRT1-mediated PCAF deacetylation mastered E2F1/P73 pathway. They not only found that SIRT1 directly represses E2F1-dependent P73 promoter activity in normal culture media and activates it in response to DNA damage but identified that the induced apoptotic DNA damage releases PCAF from SIRT1 repression. This favors the assembly of transcriptionally active PCAF/E2F1 complexes on the P73 promoter and p53-independent apoptosis (37). It is believed that E2F1 rapidly activates transient SIRT1 expression in response to cell damage for protection and to stimulate repair; this increase in SIRT1 expression impacts apoptosis *via* the E2F1 pathway (38). However, Wong et al. showed that SIRT1-mediated deacetylation of the tumor suppressor retinoblastoma protein (RB) (39), which is a direct suppressor of E2F1 transcriptional activity suggesting that SIRT1 might limit its inhibitory effect on E2F1. Jablonska et al. demonstrated that SIRT1 deacetylates Rb in the Rb/E2F1 complex, resulting in dissociation of E2F1 and promoted proliferation in oligodendrocyte progenitor cell (40). Additionally, Imperatore et al. found that SIRT1 inhibition blocks E2F1 phosphorylation and transcriptional activation of its target genes (41). Together, these findings suggest the existence of a complex and dynamic regulatory relationship between E2F1 and SIRT1 that varies according to the cellular context.

##### FOXO3a

FOXO3a promotes *SIRT1* transcription in a manner similar to that of P53. FOXO3a and P53 share two identical binding sites in the *SIRT1* promoter that impact *SIRT1* transcription. Nemoto et al. showed that acute nutritional stress through pheochromocytoma treatment caused FOXO3a to translocate into the nucleus and bind to P53, which dissociates the complex from the *SIRT1* promoter, thus eliminating the inhibitory effect on *SIRT1* (29). On the other hand, FOXO3a can be deacetylated by SIRT1, an interaction which enables the FOXO family to participate in the regulation of various biological processes such as the cell cycle, energy metabolism, and oxidative stress (42, 43).

##### C-Myc

Negative feedback loops also exist between c-myc and SIRT1, which impact multiple biological processes (44). c-myc can bind to the *SIRT1* promoter and induce *SIRT1* transcription. On the other hand, SIRT1 can directly deacetylate c-myc, which destabilizes the c-myc protein and further inhibits *SIRT1* expression (44).

### Post-Transcriptional Regulation of *SIRT1*

#### Non-Coding RNAs (NcRNAs)

SIRT1 is post-transcriptionally regulated by numerous classes of ncRNAs, especially microRNAs (miRNAs). miRNAs bind to the 3’ untranslated region (UTR) of target mRNAs, inhibiting mRNA translation or promoting mRNA degradation. Several miRNAs (miR-9, miR-34, miR-133, miR-146, and miR-199) downregulate SIRT1 protein expression (45). In addition, many circular RNAs and long ncRNAs (lncRNAs) can serve as competing endogenous RNAs, which can sponge miRNAs or proteins to regulate SIRT1 translation (46–49). Wang et al. identified an antisense (AS) lncRNA that is complementary to *SIRT1*, which competes with miR-34a for binding to the *SIRT1* 3’ UTR, affecting SIRT1 translation (50). Furthermore, Li et al. found SIRT1-AS-lncRNA could directly bind to the 3’ UTR of *SIRT1*, increasing its stability and promoting SIRT1 translation (51). Thus, *SIRT1* expression is regulated by ncRNAs.

#### The Human Antigen R (HuR) RNA-Binding Protein

The HuR protein regulates mRNA stability and translation through three RNA-recognition motifs present in the HuR protein. Abdelmohsen et al. reported that HuR could stabilize *SIRT1* mRNA by binding to its 3’ UTR, while in senescing cells, checkpoint kinase 2 (CHK2)-mediated phosphorylation dampened the interaction between HuR and *SIRT1* mRNA, leading to decreased SIRT1 protein levels (52).

## Functional Regulation of SIRT1 Protein

**Figure 4:**
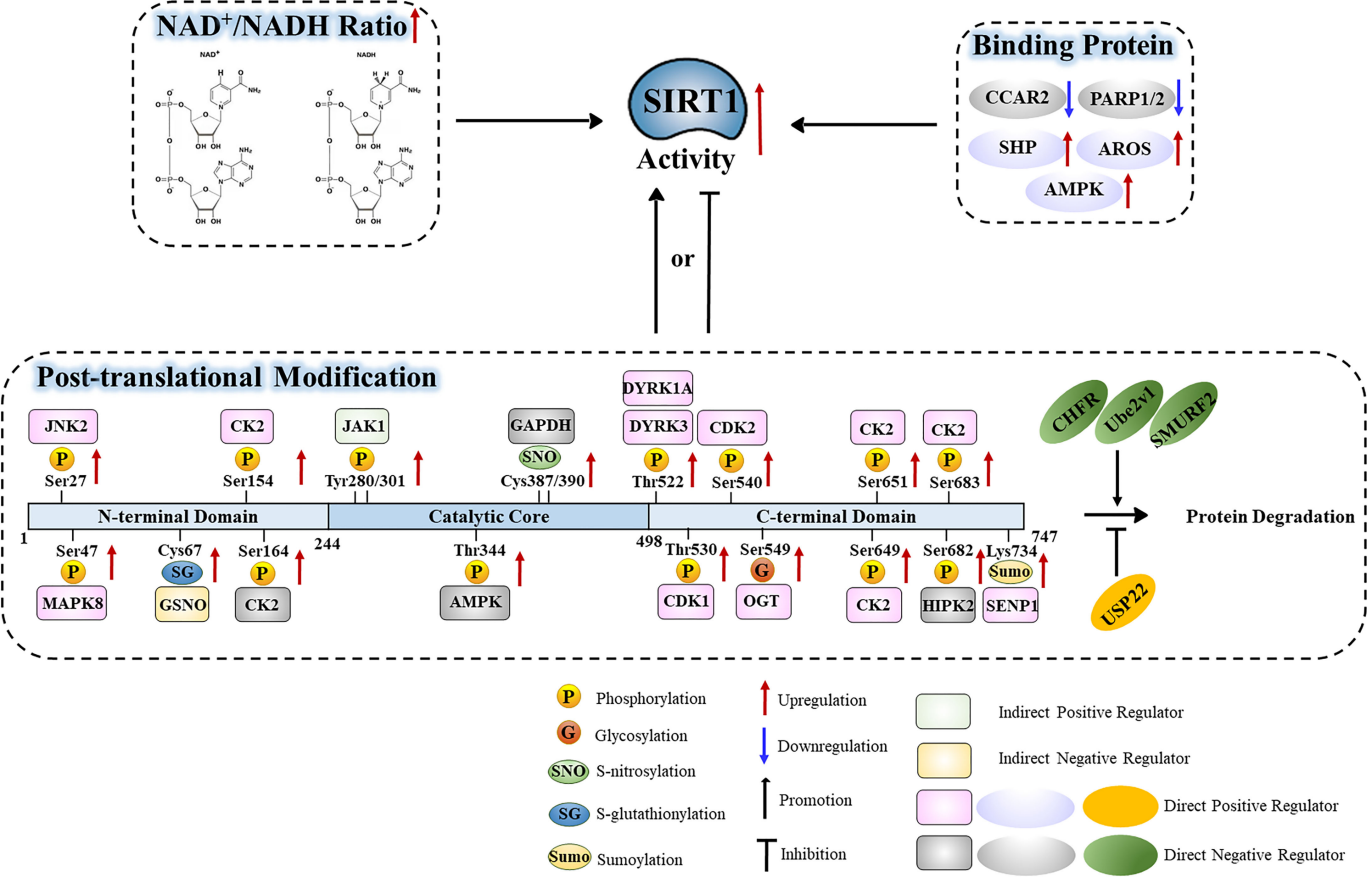
Functional regulation of the SIRT1 protein in relation to the NAD^+^/NADH ratio, binding partners, and post-translational modifications.

In addition to being regulated at the transcriptional and post-transcriptional levels, SIRT1 activity is influenced by additional factors that operate on the SIRT1 protein. The activity of SIRT1 is heavily influenced by the NAD^+^/NADH ratio, its interaction with binding partners, and its post-translational modifications. These mechanisms of SIRT1 regulation are depicted in **Table 2** and **Figure 4**.

**Table 2:** Functional regulation of SIRT1 protein.

Level of Regulation	Specific Mode		Molecules	Effect
NAD^+^/NADH Ratio	NAD^+^/NADH		NAD^+^, NADH	Upregulation
SIRT1-binding proteins	Protein interaction		CCAR2, PARP1 & PARP2	Downregulation
			AROS, AMPK, SHP	Upregulation
Post-translational modification of SIRT1	Ubiquitination		USP22	Upregulation
			CHFR, Ubc13, Ube2v1, MDM2, SMURF2	Downregulation
	Sumoylation		SENP1	Downregulation
	phosphorylation	Threonine phosphorylation	AMPK	Up/Downregulation
			CDK1, DYRKs	Upregulation
		Serine Phosphorylation	CDK1, CK2, MAPK8/JNK1, JNK2, AMPK	Upregulation
			HIPK2	Downregulation
		Tyrosine phosphorylation	JAK1	Required for SIRT1-mediated acetylation of STAT3
	Glycosylation		O-GlcNAc transferase	Upregulation
	S-nitrosylation		iNOS	Downregulation
	S-glutathionylation		GSNO	Downregulation

### NAD^+^/NADH Ratio

As an NAD^+^-dependent protein deacetylase, the deacetylase activity of SIRT1 is predominantly influenced by the cellular NAD^+^/NADH ratio such that SIRT1 activity increases as the NAD^+^/NADH ratio increases (53). Several studies have identified that in many pathophysiological processes, the NAD^+^/NADH ratio is significantly correlated with SIRT1 activity (54). Importantly, exogenously introduced NAD^+^/NADH can promote SIRT1 activity (55). Thus, the NAD^+^/NADH ratio is one of the most important regulators of SIRT1 function.

### SIRT1-Binding Proteins

#### CCAR2 Negatively Regulates SIRT1 Activity

Cell cycle and apoptosis regulator protein 2 (CCAR2, or DBC1) is the core component of the DBIRD complex, a multiprotein complex that acts at the interface between core mRNP particles and RNA polymerase II and integrates transcript elongation with the regulation of alternative splicing: the DBIRD complex affects local transcript elongation rates and alternative splicing of a large set of exons embedded in (A + T)-rich DNA regions. CCAR2 has emerged as an important player of the DNA damage response. Indeed, upon genotoxic stress, phosphorylated-CCAR2 increases its binding to SIRT1 and inhibits SIRT1 activity (56). CCAR2can block the binding of SIRT1 to its target proteins by competitively binding to the SIRT1 catalytic domain through its leucine zipper domain (57–59). CCAR2 regulates SIRT1-mediated deacetylation of P53 and FOXO3a without affecting the levels of SIRT1 itself (58, 59). Additionally, Park et al. found that CCAR2 sumoylation could enhance the interaction between CCAR2 and SIRT1, thus inhibiting transcriptional activation by P53 (60). More recently, Iqbal et al. reported that hydrogen sulfide-induced GAPDH sulfhydration leads to its redistribution into the nucleus and interaction with CCAR2 inside the nucleus, which disrupts the inhibitory effect of CCAR2 on SIRT1 and unltimately activated-SIRT1 deacetylates microtubule-associated protein 1 light chain 3 beta (MAP1LC3B/LC3B) to induce its translocation from nucleus to cytoplasm and activate autophagy (61). Together, these results indicate that CCAR2 negatively regulates SIRT1 activity.

#### PARP1 and PARP2 Negatively Regulate SIRT1 Activity

Poly ADP-ribose polymerase (PARP) is a ribozyme that mediates poly-ADP-ribosylation of proteins and plays a key role in DNA repair which consumes molecular NAD^+^. When DNA damage occurs in cells, PARP1 can recognize and bind DNA breaks within chromatin, and then recruit NAD^+^-dependent ADP ribose units, histones and related enzymes to complete the DNA damage repair process through a series of catalytic regulatory reaction (62). Bai et al. found that in brown adipose and muscle tissues, PARP1 depletion or inhibition could increase SIRT1 activity *in vivo* and *in vitro*, respectively (63). Thus, PARP1 negatively regulates SIRT1 activity in an NAD^+^-dependent manner. PARP2 deficiency also enhances SIRT1 activity, however unlike PARP1, this occurs independent of NAD^+^ level (64).

#### AROS Positively Regulates SIRT1 Activity

Active regulator of SIRT1 (AROS) encodes a nuclear protein containing 142 aa residues, which is identified as a direct interactant of SIRT1. AROS directly binds at a site (amino acids 114–217) distal to the SIRT1 catalytic domain (amino acids 245–495), and *via* this interaction promotes SIRT1 deacetylation activity (65, 66). Yeast two hybrid experiment confirmed that AROS can bind to SIRT1 to directly activate SIRT1 activity and inhibit p53-dependent transcriptional activation (65). However, it is thought that AROS is a weak activator of SIRT1, and that this form of activation is relatively non-existent in tumor cells. For example, Knight et al. found that AROS-mediated acetylation of P53 depends on the cell processing environment (67). When AROS was inhibited and SIRT1 activity persisted, AROS showed no regulatory effect on apoptosis in non-tumor cells. Nevertheless, Kokkola et al. showed that AROS is indeed a SIRT1 agonist through several *in vitro* SIRT1 activity assays (68). However, the interaction between AROS and SIRT1 was very weak, and SIRT1 activity would not be affected by AROS in non-cancerous human cells. In summary, the function of AROS in regulating SIRT1 protein expression varies across different cellular contexts.

#### AMPK Promotes SIRT1 Activity

AMP-activated protein kinase (AMPK) is an energy sensor protein kinase which can monitor changes in the level of ATP and directly phosphorylate metabolic enzymes to act as a key regulator in energy metabolism (69). AMPK promotes SIRT1 activity by increasing intracellular NAD^+^ level [58]. Interestingly, SIRT1 can indirectly activate AMPK signaling in the process of chronic metabolic adaptations (70). On the other hand, AMPK can directly mediate SIRT1 phosphorylation and promote SIRT1 deacetylase activity (71). Thus, AMPK is an important regulator of SIRT1 activity.

#### SHP Promotes SIRT1-Mediated Histone Deacetylation

The orphan nuclear receptor small heterodimer partner (SHP) protein has been identified as a co-transcriptional factor of many nuclear receptors. Chanda et al. found that SHP and SIRT1 can co-localize and interact *in vivo*, and inhibition of SIRT1 activity leads to a recovery from the intrinsic repressive activity of SHP (72). Chromatin immunoprecipitation (ChIP) experiments revealed that SHP can recruit SIRT1 to the promoters of specific target genes to mediate SIRT1 deacetylation of histones H3 and H4, thus preventing transcription of the target genes. However, there is no definitive evidence that SHP can directly activate SIRT1 activity.

### Post-Translational Modification of SIRT1

#### Ubiquitination

Ubiquitination is an important post-translational modification that regulates protein stability. In this way, ubiquitin covalently binds to target proteins through a series of reactions, which can result in degradation of the target proteins, thus impacting their stability and activity. SIRT1 ubiquitination is regulated by numerous proteins, including ubiquitin-specific peptidase 22 (USP22) and the E3 ubiquitin ligase CHFR (73, 74). SIRT1 undergoes ubiquitination in response to a variety of cellular contexts. Geng et al. found that SIRT1 ubiquitination reduces its expression during lipid metabolism in the liver (75). Additionally, the ubiquitin-conjugating E2 enzyme variant protein 1 (Ube2v1), an E2 member of the ubiquitin-proteasome system, promotes Ubc13-mediated ubiquitination and degradation of SIRT1, thereby inhibiting H4K16 acetylation and the transcription of autophagy-related genes in colorectal cancer (76). SIRT1 is also ubiquitinated by the E3 ligase MDM2 in response to DNA damage (77). Interestingly, this modification does not seem to affect SIRT1 activity or stability, but rather it modulates the nuclear translocation of SIRT1, regulating its function in the DNA damage response (77). SIRT1 also undergoes ubiquitination in colorectal cancer. Yu et al. discovered that the E3 ubiquitin ligase SMURF2 ubiquitinates SIRT1 and mediates its degradation; depletion of SMURF2 upregulates SIRT1 and induces the proliferation of colorectal cancer *in vitro* and *in vivo* (78). Together, these results suggest that ubiquitination plays a key role in regulating SIRT1 stability in response to cellular conditions such as DNA damage and cancer.

#### Sumoylation

Under normal physiological conditions, lysine 734 of human SIRT1 remains sumoylated, preserving its deacetylase activity, which permits SIRT1 to inhibit the transcription of apoptosis-related genes. Yang et al. discovered that SIRT1 could be desumoylated at lysine 734 by senstrin-specific protease 1 (SENP1), and that elevated SIRT1 sumoylation increased its stability and deacetylase activity *in vitro* (79). Interestingly, the interaction between SIRT1 and SENP1 was enhanced by oxidative stress, triggering ace-P53-dependent apoptosis (80). Together, these results suggest that sumoylation is important to the regulation of SIRT1 function.

#### Phosphorylation

##### Threonine Phosphorylation

Several kinases have been reported to catalyze threonine phosphorylation of SIRT1. AMP-activated protein kinase (AMPK) directly interacts with the core catalytic domain of SIRT1 and mediates phosphorylation of threonine 344 of SIRT1, which directly inhibits its deacetylation activity (81). Lau et al. found that AMPK-mediated threonine phosphorylation of SIRT1 can also dissociate SIRT1 from the negative regulator DBC1 and enhance P53 deacetylation (82). In addition, cyclin dependent kinase 1 (CDK1) mediates threonine 530 phosphorylation of SIRT1, increasing its activity (83). Dual specificity tyrosine-phosphorylated and regulated kinase (DYRK) is a family of highly conserved protein kinases that phosphorylate their own tyrosine sites and serine/threonine residues on exogenous substrates (84). DYRKs are pleiotropic factors that phosphorylate a broad set of proteins involved in many different cellular processes (85). DYRK1A and DYRK3 were shown to interact with SIRT1 through co-immunoprecipitation and GST pull-down assays (86). DYRK1A and DYRK3 can phosphorylate SIRT1 at threonine 522, which promotes deacetylation of p53 and inhibits apoptosis. Lu et al. demonstrated that phosphorylation of threonine 522 of SIRT1 is crucial for tissue-specific regulation of SIRT1 activity, activation of hepatic SIRT1 in response to excess caloric intake, and a threonine 522 dephosphorylation mimic impairs energy metabolism (87). In addition, Utani et al. reported that phosphorylation of human SIRT1 on threonine 530 by DYRK2 modulates DNA synthesis, preventing DNA damage upon replication stress (88). Together, these results demonstrate that threonine phosphorylation of SIRT1 is crucial for regulating its activity.

##### Serine Phosphorylation

Serine phosphorylation of SIRT1 is facilitated by several kinases and plays an important role in regulating SIRT1 function. CDK1-mediated SIRT1 phosphorylation on serine 540, and casein kinase 2 (CK2)-mediated phosphorylation on serine 154, serine 649, serine 651, and serine 683, can significantly enhance SIRT1 activity and interaction with its targets (83, 89). Furthermore, CK2-mediated phosphorylation of serine 164 of SIRT1 inhibits its deacetylase activity (90). Moreover, MAPK8/JNK1 (c-Jun N-terminal kinase 1)-mediated SIRT1 phosphorylation of serine 47 and JNK2-mediated phosphorylation of serine 27 promotes the nuclear translocation of SIRT1, enhances its stability, and promotes its deacetylation activity (91–93). Conrad et al. found that DNA damage induces an interaction between SIRT1 and homeodomain-interacting protein kinase 2 (HIPK2), which results in the phosphorylation of SIRT1 at serine 682 and lethal damage (94). DNA damage-induced SIRT1 serine 682 phosphorylation disrupts the interaction between SIRT1 and active regulator of SIRT1 (AROS), thus inhibiting SIRT1 activity. Reduced SIRT1 activity enables efficient P53 acetylation, expression of pro-apoptotic P53 target genes, and potentiation of the DNA damage-induced cell death response. Lastly, AMPK activation also increases serine 27 phosphorylation, weakening the interaction between DBC1 and SIRT1, and indirectly enhancing SIRT1 activity (71). Thus, serine phosphorylation is an important mediator of SIRT1 function.

##### Tyrosine Phosphorylation

Tyrosine 280 and tyrosine 301, which are both highly conserved sites in the catalytic domain of SIRT1, are key residues that are phosphorylated by Janus kinase 1 (JAK1). While JAK1-mediated SIRT1 phosphorylation does not alter SIRT1 deacetylase catalytic activity, it is required for SIRT1-mediated acetylation of the downstream transcription factor STAT3. Interestingly, IL-6 stimulation enhances JAK1-mediated phosphorylation of SIRT1 (95). These results suggest that tyrosine phosphorylation is a key player in regulating SIRT1.

#### Glycosylation

SIRT1 is O-GlcNacylated at serine 549 in its C-terminus, which directly enhances its deacetylase activity *in vitro* and *in vivo*. In cellular and mouse models of oxidative and metabolic stress, SIRT1 O-GlcNacylation levels and SIRT1 deacetylase activity increased, which protected cells from stress-induced apoptosis (96). However, Chattopadhyay et al. found that SIRT1 was glycosylated at its N-terminus; however, neither this modification nor its loss affected the intrinsic SIRT1 activity (97). This study demonstrated that glycosylation of SIRT1 can lead to the ubiquitin-mediated degradation of SIRT1 under nutrient-rich conditions. Nevertheless, the exact role of glycosylation in regulating SIRT1 remains unclear and requires further investigation.

#### S-Nitrosylation

Protein S-nitrosylation is a selective covalent post-translational modification that adds a nitrosyl group to the reactive thiol group of a cysteine, forming S-nitrosothiol (SNO); it is a key mechanism in transferring NO-based signaling. S-nitrosylation can regulate protein activity, stability, localization, and protein-protein interactions across myriad physiological processes (98, 99). Kornberg et al. found that s-nitrosylated GAPDH is transported into the nucleus where it physically interacts with SIRT1, transferring nitric oxide groups to cysteine 387 and cysteine 390 of SIRT1 with the help of inducible nitric oxide synthase (iNOS) (100). Nitrosylated SIRT1 (SNO-SIRT1) has restricted deacetylase activity. Furthermore, elevated levels of iNOS, in response to stress and inflammation, induce s-nitrosylation of SIRT1, which limits SIRT1 activity and restricts the inhibitory effect of SIRT1 on NF-κB and P53 signaling (101, 102). Together, these results suggest that S-nitrosylation is an emerging mechanism that controls SIRT1 activity.

#### S-Glutathionylation

S-Glutathionylation is the process of forming mixed disulfides between glutathione and cysteine residues in target proteins. SIRT1 forms mixed disulfides with S-nitrosoglutathione (GSNO)-sepharose and S-glutathiolated cysteine 67. SIRT1 activity is not directly affected by S-glutathionylation, but rather it is impacted by low concentrations of reactive glutathione (103). Interestingly, reversible S-glutathionylation of SIRT1 mediated by glutaredoxin 2 is key to the formation of a functional vascular system (104). Thus, S-glutathionylation appears to be important in the control of SIRT1 function.

## SIRT1 in the Regulation of Inflammation

### Inflammation-Induced Changes in Expression of SIRT1

SIRT1 expression varies depending upon the different immune states. A growing body of evidences suggests that SIRT1 is downregulated as part of the acute inflammatory response and in related diseases, both *in vivo* and *in vitro* (105–107). In accordance with these findings, some drugs and upstream molecules exhibit great anti-inflammatory activity by upregulating SIRT1 expression (108–112). IFN-γ disrupts energy expenditure and metabolic homeostasis in chronic inflammation of skeletal muscle cells by inducing HIC1 and transcriptional modulator class II transactivator (CIITA) (113). Liu et al. demonstrated that SIRT1 increases during TLR4-induced endotoxin tolerance and represses TLR4-induced TNF-transcription in normal and endotoxin-tolerant THP1 cells (114). Moreover, Kong et al. identified circ-SIRT1 as a novel suppressive regulator of the inflammatory phenotype of vascular smooth muscle cells (VSMCs) by virtue of its binding to miR-132/212, which interferes with the SIRT1 3’ UTR, ultimately promoting SIRT1 upregulation (115). However, elevated SIRT1 levels have also been reported. For example, in LPS-stimulated H9c2 cardiomyocytes, SIRT1 mRNA and protein levels were both significantly upregulated, and cardiac tissues harvested from sepsis mice in the cecal ligature and puncture (CLP) model showed that SIRT1 expression was increased in sepsis (116). Some studies have suggested that there is no relationship between the inflammatory state and SIRT1 expression. For example, Orecchia et al. reported that while SIRT1 significantly diminished the inflammatory responses to IL-1β and TNF-α in human dermal microvascular endothelia cells (HDMEC), no significant change in SIRT1 expression was observed in psoriatic HDMEC treated with IL-1β, IFN-γ, IL-17, or VEGF-A (117). Nakamura et al. also confirmed the suppressive role of SIRT1 in decreasing macrophage activation (*iNOS*, *IL-1β*, *MCP1*, *CCL5*, *CXCL10*, *GzmB*) using LPS-stimulated bone marrow-derived macrophage (BMDM) models; SIRT1 expression changed very little in LPS-stimulated BMDM (118).

### Variation in SIRT1 Activity Associated With Inflammation

Understandably, the activity of SIRT1 is crucial for its effectiveness in regulating inflammation. There is mounting evidence showing that SIRT1 activity is hindered in hyper-inflammation and related organ injuries. As mentioned earlier, dynamic variation of the NAD^+^/NADH ratio is a prerequisite for changes in SIRT1 activity in inflammation; SIRT1 activity and cellular NAD^+^ levels simultaneously decrease while proinflammatory gene expression increases in alcohol-induced inflammation and oxidative stress (119). Liu et al. showed that NAD^+^ informs SIRT1, directing a sequential epigenetic switch between early and late TLR4 responses in a THP-1 promonocyte sepsis cell model and in human sepsis blood leukocytes (114). They further demonstrated that SIRT1 and SIRT6 couple a switch from increased glycolysis to increased fatty acid oxidation as early inflammation transitions to late inflammation, which requires NAD^+^ production using nicotinamide phosphoribose transferase (120). In addition, Khadka et al. found that hyperactivation of PARP-1 reduces the NAD^+^/NADH ratio resulting in a decrease in SIRT1 activity (121). Furthermore, enzymatic action of NAD(P)H quinone oxidoreductase 1 can attenuate adriamycin-induced cardiac inflammation and related dysfunction by elevating the NAD^+^/NADH ratio, recovering SIRT1 activity. Finally, oxidative stress can be accompanied by a decrease in the NAD^+^ level and SIRT1 activity (122). Accordingly, ROS could directly oxidize the cysteines residues of SIRT1 inhibiting its activity (123). Choi et al. indicated that CK2-mediated SIRT1 phosphorylation at ser164 inhibits SIRT1 enzymatic activity in inflammatory pathologies like nonalcoholic fatty liver disease (90). The application of special agonists of SIRT1, which directly upregulate SIRT1 activity, invariably attenuates inflammatory responses and related tissue injuries (111, 124–126). These studies indicate that SIRT1 deacetylase activity definitely endows the protein with anti-inflammatory activity.

### Mechanisms of SIRT1 in Regulating Inflammation

Inflammation is an automatic host defense mechanism that responds to both infection and non-infectious factors. Inflammatory responses are usually beneficial to the elimination of pathogens but can sometimes result in tissue damage. Inflammatory cytokines including IL-1β, IL-6, TNF-α, and CCL2 are key players in the regulation of inflammation. If local inflammation is not effectively controlled, inflammatory reactions can spread throughout the whole body, and inflammatory factors can damage tissues. Further, an imbalance between pro-inflammatory and anti-inflammatory cytokines can amplify inflammation, thus promoting a vicious circle of inflammation and damage. Studies suggest that SIRT1 has strong anti-inflammatory effects and can alleviate injuries sustained as a result of an over-active immune system by inhibiting the expression factors involved in inflammatory pathways such as NF-κB, HIF1α, activator protein 1 (AP-1), and P38MAPK (127), which will be discussed in more details in this section.

#### SIRT1 Regulates Inflammatory Cytokine Expression Through Histone Deacetylation

As previously mentioned, SIRT1-mediated deacetylation of histones in the promotor region of target genes directly inhibits target gene transcription, a mechanism though which SIRT1 suppresses inflammatory cytokine expression. In the endotoxin tolerance process of sepsis, SIRT1 accumulates in the promoters of IL-1β and TNF-α and NAD^+^ levels increase, which enhances H3K16 deacetylation (128). Zhang et al. confirmed that SIRT1 can reduce histone H3K9 acetylation in the promoters of IL-6 and TNF-α, blocking their expression (15). Moreover, Chen et al. demonstrated that SIRT1 targets the TNF-α promoter, reducing H3K16 acetylation and inhibiting TNF-α transcription during sepsis-induced inflammation (16).

#### SIRT1 Regulates Inflammation Though Different Signaling Pathways

##### NF-κB

Activation of NF-κB signaling plays an important role in sepsis-induced inflammation and is one of the most widely studied inflammatory pathways. NF-κB is composed of NF-κB1 (P105 and P50), NF-κB2 (P100 and P52), P65, RELB, and c-REL (129). In quiescent conditions, NF-κB normally exists as a component of inactive cytoplasmic complexes bound by members of the inhibitor of κB (IκB) family. When inflammatory responses are activated, IκB is degraded owing to IκB kinase (IKK)-mediated phosphorylation. In turn, NF-κB is translocated to the nucleus and activates gene transcription involved in the establishment of immune and inflammatory responses (130). SIRT1 can directly inhibit inflammatory gene expression, for example, Yeung et al. demonstrated that SIRT1 deacetylates the NF-κB P65 subunit, inhibiting NF-κB activity (131). Subsequent studies have confirmed that SIRT1 inhibits NF-κB-mediated inflammatory cytokine expression by downregulating acetylation of P65 through its deacetylation at lysine310, resulting in anti-inflammatory effects (21). In addition, acetylation of P65 at lysine 310 affects the methylation of lysine 314 and lysine 315 by SET9, accelerating the ubiquitination and degradation of P65 (132). SIRT1 influences the nuclear translocation of NF-κB and its DNA binding ability. Lei et al. found that resveratrol, a classic SIRT1 agonist, inhibits nuclear aggregation of P65 and thus its DNA binding ability (133). During inflammation, SIRT1 is recruited to the transcriptional regulatory regions of NF-κB targets (127). Liu et al. demonstrated that long-term LPS treatment promotes SIRT1 accumulation in the promoters of inflammatory cytokines and deacetylation of P65 (114). Elevated NAD^+^ levels, along with SIRT1 and RelB accumulation at the TNF-α promoter, blocked TNF-α transcription. SIRT1 can also inhibit IκB degradation, alleviating LPS-induced inflammation in macrophages (134).

Alternatively, SIRT1 can indirectly inhibit NF-κB signaling by regulating the expression of mediator proteins such as AMPK and PPARs. The interaction between SIRT1 and AMPK plays an important role in the inflammatory response. AMPK is an important inhibitor of NF-κB, and SIRT1 can activate AMPK, which can indirectly affect NF-κB (135). Additionally, Planavila et al. found that SIRT1 overexpression enhances the interaction between PPARα and P65, inhibiting the activation of NF-κB, and thus suppressing transcription of the inflammatory cytokine MCP-1 (136). Thus, the effects of SIRT1 on NF-κB renders SIRT1 important to the study of inflammatory diseases including sepsis. Although SIRT1-mediated deacetylation of P65 on lysine 310 has been well studied, the selective regulation of NF-κB-related molecules by SIRT1 requires further exploration. Moreover, there exists relevant evidence indicating NF-κB might inhibit SIRT1 signaling. Voelter-Mahlknecht et al. isolated and characterized human *SIRT1*, revealing a promotor containing a number of NF-κB binding sites through characterization of the 5’ flanking genomic region (137). Zhang et al. showed that overexpression of NF-κB P65 significantly upregulated SIRT1 mRNA and protein levels, and P65 knockdown inhibited TNF-α-stimulated SIRT1 expression (138). Katto et al. performed an electrophoretic mobility shift assay affirming the direct binding of NF-κB to the SIRT1 promoter (139). However, the specific mechanism of NF-κB regulation of SIRT1 still demands further exploration.

##### HIF1α

HIF1α is also a key transcriptional factor in oxidative stress and pro-inflammation responses. Lim et al. first reported that SIRT1 directly interacts with HIF1α mediating the deacetylation of HIF1α at Lys374. Through this interaction, SIRT1 inactivates HIF1α by blocking the recruitment of P300 acetyltransferase in hypoxia (140). Furthermore, the direct deacetylation by SIRT1 on HIF1α is required for HIF1α protein stability during hypoxia (141). Consistently, SIRT1 suppresses the high expression of proinflammatory factors, including IL-6, IL-8, and TNF-α, and alleviates intestinal epithelia barrier dysfunction by hindering the expression and activity of HIF1α in necrotizing enterocolitis (142). Liu et al. demonstrated that SIRT1-HIF1α signaling contributes to the prerequisite role of SIRT1 in orchestrating the balance between proinflammatory T helper type 1 cells and anti-inflammatory Foxp3(+) regulatory T cells in dendritic cells (143). Glucose metabolism also greatly contributes to immunologic homeostasis of immune cells. SIRT1 can alleviate allergic airway inflammation by negatively regulating mTOR and HIF1α signaling coupled with glycolytic metabolism and suppressing HIF1α-targeting of IL-9 production of Th9 cells (144).

##### AP-1

The AP-1 transcription factor is a heterodimer formed by c-Jun and c-Fos that is an important regulator of inflammation and immunity. Activated AP-1 promotes the transcription of various inflammatory factors, such as IL-2, IL-8, and TNF-α (145). SIRT1 mediates deacetylation of AP-1 by directly binding c-Jun *via* its C-terminus, impairing its transcriptional activity, and downregulating proinflammatory cytokines (146). Zhang et al. confirmed that SIRT1 can inhibit P300-mediated AP-1 acetylation and decrease cyclooxygenase 2 (COX2) transcription in macrophages (147). These results suggest that SIRT1 functions to alleviate inflammation by inhibiting the AP-1 signaling pathway.

##### P38MAPK

P38MAPK signaling pathway is activated to catalyse the reversible phosphorylation of proteins in response to inflammation (148). The mechanisms underlying the regulation of SIRT1 by P38MAPK during inflammatory responses remain largely unknown. A few studies have shown that SIRT1 can inhibit P38MAPK phosphorylation and activation (149). For example, Bi et al. showed that the SIRT1 agonist resveratrol reduced TNF-α and nitric oxide secretion by suppressing P38MAPK phosphorylation in LPS-induced inflammation of microglia (150). However, Yang et al. demonstrated that SIRT1 overexpression increased P38MAPK phosphorylation and, conversely, SIRT1 knockdown decreased P38MAPK phosphorylation in hepatic inflammation induced by ischemic injury *in vivo* and *in vitro* (128). While these initial findings show promise, the regulation of SIRT1 by P38MAPK during inflammatory responses requires further characterization.

### SIRT1-Based Therapeutics for Inflammatory Diseases

The results of various studies suggest that SIRT1 could be a novel target for systemic and tissue-specific inflammatory disease therapies.

#### Sepsis

Sepsis refers to an unbalanced systemic inflammatory reaction associated with severe injury or infection. Sepsis-associated multiple organ dysfunction syndrome (MODS) and septic shock severely reduce the survival of patients (151–153). Accumulating studies have confirmed the protective role of SIRT1 in treatment of sepsis. Sepsis is characterized by progressive sequential reactions from early-hyperinflammation to late-immunosuppression (154). SIRT1 can regulate immunometabolic polarity during the hyper-inflammatory and hypo-inflammatory phases of sepsis (155). On one hand, SIRT1 can overwhelmingly alleviate septic inflammation and related organ injuries. For example, Khader et al. found that SRT1720 treatment dramatically suppressed proinflammatory cytokines release and inflammasome activation, and ameliorated multiorgan injuries in CLP mice (156). Furthermore, Bai et al. showed that SIRT1 hindered systemic inflammation and related multiorgan injuries in myeloid-specific SIRT1 knockout mice (105). On the other hand, SIRT1 regulation of the immunosuppression phase of sepsis remains uncertain. Martin et al. showed that treatment of CLP mice with EX527 concordantly reversed immune tolerance splenic dendritic and antigen-specific tolerance of splenic CD4+ and CD8+ T cells. In addition, SIRT1 inhibition significantly decreased the ratio of CD4+ T_Reg_ repressor to CD4+ activator T cells (157). Accordingly, SIRT1 inhibition during the immunosuppression phase significantly rescued CLP mice from septic death (158). Taken together, SIRT1 is presumed to be a promising novel target for treating sepsis, but further studies are necessary to evaluate systemic immune homeostasis.

#### Liver

A growing body of evidence suggests that SIRT1 contributes greatly to protection of liver inflammation and related injuries (159–161). For instance, Yin et al. found that mice with liver-specific deletion of SIRT1 were hypersensitive to ethanol challenge. Hepatic deletion of SIRT1 promotes steatosis, inflammation, and fibrosis. They declared that SIRT1 alteration of lipin-1 mRNA splicing contributes to development of alcoholic steatosis and inflammation, which could be developed to extend therapies on alcoholic fatty liver disease (162). Another study demonstrated that hepatic stellate cells (HSC)-specific SIRT1 knockout mice were more susceptible to long-term chronic-plus-multiple binges of ethanol-induced liver fibrosis (160). In contrast, Isaacs-Ten et al. found that SIRT1 promotes increased liver inflammation and injuries post-LPS/GalN and bile duct ligation, associated with elevated activation of inflammasomes in macrophages (163). These contradictory findings suggest that SIRT1 might be a promising target for therapeutic intervention in hepatic inflammatory diseases, but further investigation is required.

#### Lung

In chronic obstructive pulmonary disease (COPD), SIRT1 activity and nuclear expression are decreased, promoting NF-κB direction to the IL-8 gene promoter (164). Yanagisawa et al. elucidated that the serum SIRT1 level was decreased in patients with COPD and that this might serve as a potential biomarker for certain disease characteristics of COPD (165). SIRT1 was observed to redress the imbalance of tissue inhibitor of matrix metalloproteinase-1 and matrix metalloproteinase-9 in the development of mouse emphysema and human COPD (166). Furthermore, Wu et al. found that SIRT1 deficiency aggravated lung vascular leakage and inflammation following particulate matter exposure. In addition, SIRT1 shows a strong protective effect against lung coagulation (167). Consistently, application of SIRT1 agonists, such as SRT1720 and resveratrol, have exhibited beneficial effects in asthmatics by suppressing inflammation (125). Fu et al. illustrated that intratracheal administration of SRT1720 significantly attenuated LPS-induced acute lung injury (ALI) and lung hyper-permeability, whereas intratracheal administration of the selective SIRT1 inhibitor EX527 aggravated LPS-induced ALI (168). Resveratrol inhibited oxidative stress and reversed the methamphetamine (MA)-stimulated higher permeability and apoptosis of alveolar epithelium (169).

#### Kidney

SIRT1 is a promising target in the treatment of kidney diseases (170). Gao et al. showed that SIRT1 deletion led to enhanced inflammation and aggravated LPS-induced acute kidney injury (AKI) (171). SIRT1 attenuated sepsis-induced AKI *via* the deacetylation of Beclin1 at lysine 430 and lysine 437, associated with autophagy (171). Likewise, SIRT1 activation by SRT1720 downregulated renal proinflammatory cytokines and hindered the infiltration of macrophages in cisplatin-induced damaged kidneys (172). Additionally, SIRT1 alleviated tubular damage during AKI *via* deacetylation of HMGB1 (173) and protected tubular cells against apoptosis *via* deacetylation of FOXO3 (174). Nguyen et al. found that SIRT1 intervention alleviated renal lipid content, oxidative stress, and inflammation, but no significant change was observed in albuminuria levels. They suggested that SIRT1-targeted therapy could ameliorate some pathological mechanisms of kidney programming due to maternal obesity, but this may not be sufficient to prevent resulting chronic kidney injury (175).

## Conclusions

SIRT1 is an NAD^+^-dependent deacetylase that exhibits decreased expression and activity during inflammation. Owing to its apparent importance to the inflammatory response, additional studies that explore the mechanisms that regulate SIRT1 expression and activity are required. Numerous transcriptional and post-transcriptional mechanisms are involved in the regulation of SIRT1 expression under different conditions. SIRT1 activity is primarily regulated by factors such as the NAD^+^/NADH ratio, SIRT1-binding proteins, and post-translational modifications. SIRT1 functions under a wide range of activity levels and is involved in determining cellular phenotypes and the activity of several signaling pathways. During inflammation, SIRT1 exerts its function through deacetylation of the NF-κB, AP-1, and P38MAPK pathways. While SIRT1 is considered to be an important molecular switch that regulates inflammation, many knowledge gaps remain in this field that require further research, including mechanisms to promote SIRT1 function for the treatment of inflammatory diseases and the clinical application of SIRT1-targeted therapies.

## Author Contributions

The manuscript was written by YY. YL and YW contributed equally to this work. All authors contributed to the article and approved the submitted version.

## Funding

Research in the authors’ laboratory is supported by the National Natural Science Foundation of China [81530064], National Natural Science Foundation of China [81773071], National Natural Science Foundation of China [81972226] and Natural Science Foundation of Shaanxi Province [2021JM-249].

## Conflict of Interest

The authors declare that the research was conducted in the absence of any commercial or financial relationships that could be construed as a potential conflict of interest.

## Publisher’s Note

All claims expressed in this article are solely those of the authors and do not necessarily represent those of their affiliated organizations, or those of the publisher, the editors and the reviewers. Any product that may be evaluated in this article, or claim that may be made by its manufacturer, is not guaranteed or endorsed by the publisher.

## Abbreviations

SIRT1, Silent information regulator sirtuin 1; SIRTs, Sirtuins; HDACIII Histone deacetylase III; FOXO1/3/4, Forkhead-box transcription factor 1/3/4; HSF1, Heat shock factor 1; HIF-1α, Hypoxia-inducible factor 1 alpha; NF-κB, Nuclear factor-kappa B; PPARα/γ , Peroxisome proliferator-activated receptor α/γ MyoD, Myoblast determination protein; ATG , Autophagy-related protein; LKB1, Liver kinase B1; DNMT, DNA methyltransferase; HIC1, Hypermethylin cancer 1; CTBP1, C-terminal binding protein 1; miRNAs, MicroRNAs; , Long non-coding RNAs; CHK2, Checkpoint kinase 2; USP22, Ubiquitin-specific peptidase 22; Ube2v1, Ubiquitin-conjugating E2 enzyme variant proteins 1; Ube2v1, Cell cycle and apoptosis regulator protein 2; AROS , Active regulator of SIRT1; SHP, Small heterodimer partner; PARP1, Poly ADP-ribose polymerase 1; SENP1, Senstrin-specific protease 1; CDK1, Cyclin dependent kinase 1; GST, Glutathione S transferase; CK2, Casein kinase 2; HIPK2, Homeodomain interacting protein kinase 2; JAK1, Janus kinase 1; iNOS, Inducible nitric oxide synthase; ChIP, Chromatin immunoprecipitation; PARP, Poly ADP-ribose polymerase; AMPK, AMP-activated protein kinase; COX2, Cyclooxygenase 2; IκB, Inhibitor of κB; IKK, IκB kinase; CLP, Cecal ligature and puncture; BMDM, Bone marrow-derived macrophage; COPD, Chronic obstructive pulmonary disease; AKI, Acute kidney injury.
